# Penetration of anticancer drugs through tumour tissue as a function of cellular packing density and interstitial fluid pressure and its modification by bortezomib

**DOI:** 10.1186/1471-2407-12-214

**Published:** 2012-06-06

**Authors:** Rama H Grantab, Ian F Tannock

**Affiliations:** 1 Divisions of Research and of Medical Oncology and Hematology, Princess Margaret Hospital and University of Toronto, Toronto, Canada; 2 University of Toronto, Toronto, Canada

## Abstract

**Background:**

Limited penetration of anticancer drugs in solid tumours is a probable cause of drug resistance. Our previous results indicate that drug penetration depends on cellular packing density and adhesion between cancer cells.

**Methods:**

We used epithelioid and round cell variants of the HCT-8 human colon carcinoma cell lines to generate tightly and loosely packed xenografts in nude mice. We measured packing density and interstitial fluid pressure (IFP) and studied the penetration of anti-cancer drugs through multilayered cell cultures (MCC) derived from epithelioid HCT-8 variants, and the distribution of doxorubicin in xenografts with and without pre-treatment with bortezomib.

**Results:**

We show lower packing density in xenografts established from round cell than epithelioid cell lines, with lower IFP in xenografts. There was better distribution of doxorubicin in xenografts grown from round cell variants, consistent with previous data in MCC. Bortezomib pre-treatment reduced cellular packing density, improved penetration, and enhanced cytotoxcity of several anticancer drugs in MCC derived from epithelioid cell lines. Pre-treatment of xenografts with bortezomib enhanced the distribution of doxorubicin within them.

**Conclusions:**

Our results provide a rationale for further investigation of agents that enhance the distribution of chemotherapeutic drugs in combination with conventional chemotherapy in solid tumours.

## Background

Solid tumours have a complex microenvironment that includes malignant cells, several types of normal cells and an extracellular matrix (ECM), all of which may influence sensitivity to anticancer drugs. In order for a drug to be effective, it must be delivered through the tumour’s tortuous and leaky vasculature, cross vessel walls into the interstitium, and penetrate multiple layers of cells to reach all of the cancer cells in a cytotoxic concentration. Limited distribution of several chemotherapeutic agents has been shown in multi-cellular models in tissue culture and in experimental and human tumours and is a probable cause of clinical drug resistance [1-8].

Multilayered cell cultures (MCC) can be established by growing tumour cells on collagen-coated microporous Teflon membranes, and have been used to quantify tissue penetration of anticancer drugs [3,6,9,10]. MCC can be grown from various tumour cell lines, have a symmetrical planar structure, and an ECM similar (though not identical) to corresponding tumours grown *in vivo*[9-11]*.* Using MCC established from colon carcinoma cell lines with differences in cellular adhesion and packing density, we observed greater penetration and cytotoxicity of anticancer drugs in loosely packed MCC [3]. Improved tissue penetration of paclitaxel and doxorubicin has been observed in tumour histocultures and xenografts following pre-treatment that induced apoptosis and reduced tumour packing density [12-14]. Pre-treatment with anti-adhesive agents, such as hyaluronidase or antibodies targeted to cellular adhesion molecules, can also enhance sensitivity of solid tumours to chemotherapeutic drugs by disrupting cell-cell adhesion [15,16].

Pre-clinical and clinical studies have shown that inhibition of the 26S proteasome may enhance sensitivity to chemotherapy and radiation therapy [17-20]. The 26S proteasome is a large multi-catalytic structure responsible for the degradation of cellular proteins involved in cell cycle progression, cell survival, transcriptional activity, and cell signalling. The proteasome inhibitor bortezomib, approved for the treatment of multiple myeloma, has been shown to inhibit growth of some solid tumours [21-23]. Bortezomib can disrupt cell-cell adhesion in multi-cellular spheroids derived from prostate and ovarian cancer cells, and its efficacy in multilayer systems is similar to or greater than that observed in monolayers [24]. Pre-treatment with bortezomib has been shown to enhance cytotoxicity of conventional anticancer drugs for solid tumours, including irinotecan in colon carcinoma xenografts, and gemcitabine in non-small cell lung carcinoma xenografts [17,18]. Bortezmib’s mechanism of action in solid tumours is uncertain, but its ability to enhance effects of chemotherapy and radiation therapy may be due to inhibition of cell-adhesion mediated drug resistance (CAM-DR) through effects on the tumour microenvironment [24]. Bortezomib also inhibits angiogenesis in prostate and pancreatic cancer xenografts [19,25], and alters tumour response to hypoxia, by suppression of HIF-1α, in cervical carcinoma xenografts and human colorectal cancer [26].

The identification of microenvironmental factors that impair drug transport is instrumental in the development of agents that can modify the tumour microenvironment to enhance chemotherapeutic efficacy. The present study uses MCC and tumour xenografts, derived from established human colon carcinoma cell lines, to address the hypothesis that limited drug penetration in tumour xenografts can decrease chemotherapeutic cytotoxicity and that modification of the tumour environment by bortezomib might improve the penetration of anti-cancer drugs through tumour tissue.

## Methods

### Cell lines

Experiments were undertaken using the HCT-8Ea and HCT-8E11 human colon carcinoma cell sub-lines which have usual epithelioid phenotypes. The HCT-8 E11 and Ea sublines are hemizygous for the α-E-catenin gene (*CTNNA1*). A transition from the eipthelioid HCT-8 E11 subline to the round morphotype HCT-81R1 sublines is due to a mutation in the second allele of *CTNNA1I* and loss of adherens junctions. Although the HCT-8Ra sublines have been shown to express α-E-catenin, they fail to form tight intracellular junctions [27]. The HCT-8Ea and HCT-8Ra cell lines were provided by Dr. W.R. Wilson (University of Auckland, New Zealand) and the HCT-8E11 and HCT-81R1 cell lines by Dr. M. Bracke (Ghent University Hospital, Ghent, Belgium); these cells were grown respectively as monolayers in α-MEM (Gibco, Burlington, ON, Canada) or RPMI medium (Gibco, Burlington, ON). Media were supplemented with 10% foetal bovine serum (FBS; Hyclone, Logan, Utah) and cultures were maintained at 37 °C in a humidified atmosphere of 95% air plus 5% CO_2_. Cells were re-established from frozen stock every ~4 months and assessed periodically for the presence of mycoplasma.

### Drugs and reagents

Ethylene glycol tetra-acetic acid (EGTA) was purchased from Sigma Chemicals and bortezomib was kindly provided by Millennium Pharmaceuticals (Cambridge, Massachusetts). 6-[^3^H]-5-fluorouracil (specific activity 10 μCi/mmol) was purchased from Moravek Biochemicals Inc. (Brea, MA). [^3^H]-gemcitabine (specific activity ^14^Ci/mmol) and [^14^C]-doxorubicin (specific activity 25 μCi/mmol) were purchased from Amersham Pharmacia Biotech (Amersham, UK), and [^14^C]-sucrose (specific activity 50 μCi/mmol) was obtained from Perkin Elmer Life Sciences Inc. (Boston, MA). Unlabeled doxorubicin (Pharmacia, Mississauga, ON, Canada), gemcitabine (Eli Lily, Toronto, ON, Canada), and 5-fluorouracil (Mayne Pharma, Montreal, PQ, Canada) were obtained from the Princess Margaret Hospital Pharmacy as their clinical formulations.

### Growth and characterization of MCC

Semi-porous Teflon membrane culture inserts (Millipore, Bedford MA) were coated with Collagen Type III as described previously [28]. Exponentially-growing cells were allowed to attach for 4-8 h and the membranes were then submerged in a large volume of stirred α-MEM (HCT-8 Ea sublines) or RPMI (HCT-8E11 sublines) medium containing 1 mM pyruvate, supplemented with 10% FBS, and allowed to grow for 5-7 days at 37 °C. Uniformity of MCC growth was assessed using a light microscope, and only MCC with uniform growth were used in experiments. To determine the number of cells in MCC, one or more of them was selected at random, trypsinized, and the cells counted using a Coulter counter.

To characterize MCC, they were fixed in 10% neutral buffered formalin for 24 h and then processed through graded concentrations of ethanol, placed in xylene overnight and then embedded in paraffin. Four-micron sections were stained with haematoxylin and eosin, or with DAPI to quantify packing density. Multiple images from each DAPI-stained MCC section were acquired using an Olympus Upright microscope with a Photometrics Coolsnap HQ2 camera; the number of nuclei per unit surface area was then quantified in each image using Media Cybernetics Image Pro PLUS software. Packing density is presented as percentage nuclear area for all cell lines and treatment conditions without taking into account differences in nuclear size. In order to ensure that changes in packing density were not isolated to the surface of MCC, packing density analysis was conducted by subdividing the MCL into 3 regions horizontally: the surface of the MCC, the middle, and the bottom of the MCC adjacent to the Teflon membrane.

### Penetration of anticancer drugs in MCC

The penetration of anticancer drugs through MCC was determined after pre-treatment with either the calcium-chelating agent EGTA to inhibit cell adhesion, or with bortezomib. MCC derived from HCT-8Ea and E11 cell lines were pre-treated with 5, 10, or 50 mM of EGTA for 30, 60, or 90 min in order to determine optimal treatment conditions for inducing change in packing density with little or no toxicity (as determined by clonogenic assays). For studies with bortezomib, MCC were incubated for 24 h with 1 μM bortezomib (this dose was selected based on the maximum reduction of packing density with minimum cytotoxicity as determined by clonogenic assays), and then incubated with PBS for 30-60 min to wash away residual drug.

To study penetration of radio-labelled anticancer drugs through MCC, they were dissolved in 2 × α-MEM and mixed in a 1:1 ratio with 1% agar solution to prevent convection. A volume of 0.5 mL was added to one side of the MCC (compartment 1), and initial drug concentrations (using a combination of radio-labelled and unlabelled drug) were: 10 μM doxorubicin, 77 μM 5-fluorouracil and 100 μM gemcitabine. These concentrations approximate those achieved in serum after *in vivo* administration and permit sensitive detection of the drug in compartment 2, which contained 18 mL of stirred culture media. Experiments were conducted at 37 °C in vials exposed to 95% air/5% CO_2_. The penetration of drug through the MCC as a function of time was assessed by liquid scintillation counting of samples withdrawn from compartment 2, and is presented as a ratio of C/C∞, where C is the measured drug concentration and C∞ is the calculated drug concentration at equilibrium between the two drug compartments. [^14^C]-sucrose was included at a concentration of 3 μM in all experiments as an internal standard, with the exception of those conducted with [^14^C]-doxorubicin. Only MCC with a maximum variation of ±20% in sucrose penetration for a given experimental condition were used in data analysis. Experiments were conducted 3-6 times with MCC ranging in cell number from 3-5 × 10^6^. Drug penetration at 6 h was calculated as the ratio of the concentration of drug in compartment 2 in treated MCL to the concentration of the drug in compartment 2 in non-treated MCL.

### Clonogenic assays

Cytotoxicity of bortezomib in monolayers or MCC was assessed alone or in sequence with doxorubicin or gemcitabine. Cultures were exposed to varying concentrations of bortezomib for 24 h, and washed in PBS for 30-60 min. After treatment, MCC were disaggregated with trypsin and washed. Serial dilutions were plated in 5 mL media, incubated for 10-14 days at 37 °C in 95% air/5%CO_2_, and stained with methylene blue. Colonies containing more than about 50 cells were counted, and surviving fraction was calculated as the ratio of mean number of colonies after treatment to the mean number of colonies for the control condition: data are presented as means and standard errors for at least 3-replicate experiments. Comparisons between treatment and control conditions were analyzed using t-tests and the mean cell survival is presented as the ratio of colonies at a particular drug concentration to colonies in the untreated condition. A one way ANOVA was conducted to test for differences in mean cellular packing density between these horizontal regions using Microsoft Excel 2007. Statistical comparisons to assess treatment efficacy in clonogenic assays were conducted using one way analysis of variance (ANOVA) followed by the Newman-Kleus post-hoc test (PRISM v5, GraphPad Inc., San Diego, USA).

### Xenografts

Six to eight week-old Swiss male nu/nu mice were housed five per cage in the animal colony at Princess Margaret Hospital, and were provided with sterile water and food ad libitum. All procedures were approved by the Institutional Animal Care Committee. Tumours were generated by injecting 10^6^ exponentially growing cells from each cell line subcutaneously into the flanks of mice. Animals were divided randomly into groups; those receiving drug treatment were injected i.p. with 0.5 or 1.0 mg/kg of bortezomib. Control animals were injected with equal volumes of saline.

To evaluate cellular packing density, mice were killed humanely 24 or 72 h after bortezomib treatment. Tumours were excised, fixed in formalin and embedded in paraffin. Packing density was assessed in 5 μm DAPI-stained sections using the procedure described above for MCC. Microvascular density (MVD) was assessed by selecting areas of interest that contained CD31^+^ regions surrounded by tumour cells with nuclear staining. MVD was assessed as percentage positive CD31^+^ staining (pixel intensity 255) per unit area using Media Cybernetics Image Pro Plus Software (Version 6.0).

To evaluate effects of drugs to delay tumour growth, mice bearing palpable HCT-8 Ea and E11 tumours (~5 mm in diameter) were divided randomly into treatment groups. Treatments were administered i.p. and consisted of either 1 mg/kg of bortezomib, 8 mg/kg of doxorubicin, 1 mg/kg of bortezomib followed 72 h later by 8 mg/kg of doxorubicin, or saline. The dose of bortezomib and the 72-h time point were chosen based on a prior study showing that these conditions lead to increased apoptosis (Ling et al., 2003), and on our experiments showing that they lead to reduced interstitial fluid pressure. Mice bearing HCT-8 1R1 and Ra tumours were randomly divided into treatment groups and administered 8 mg/kg of doxorubicin i.p or saline. Tumour diameter was measured every second day up to 9 days. Tumour measurements were converted to tumour volume (V) using the formula: V = W^2^ × Y/2; where W and Y are the smaller and larger perpendicular diameters respectively. T-tests were used to assess the differences in mean tumour volume after doxorubicin treatment between tumours derived from HCT-8 E and R subtypes and also between bortezomib pre-treated and non-treated xenografts (p < 0.05 was considered statistically significant). Differences between tumour volume in various treatment groups were assessed using tumour volume data from day 11 using a *t*-test.

### Measurement of interstitial fluid pressure

Interstitial fluid pressure (IFP) was measured in tumours 24 or 72 h after bortezomib injection using the wick-in-needle technique [29]. Measurements were conducted in anaesthetized animals with tumours ranging from 7-10 mm in diameter. The “wick”, a multi-filamentous cotton thread, was placed in the distal portion of a 23-guage needle with a custom-ground 1-2 mm side port. The needle was connected to a pressure transducer (model P23XL, Viggo-Spectramed, Oxnard CA) and an electronic data acquisition and recording system (Model MP100, World Precision Instruments, Sarasota, FL) via polyethylene tubing (Becton Dickinson, Franklin Lakes, NJ). The system was calibrated before each experiment by varying the position of the needle tip a known distance above or below a reference elevation. The entire system was flushed with heparin sulfate/saline solution (1:10) prior to and following each measurement. T-tests were used to assess the differences in mean IFP after between tumours derived from HCT-8 E and R subtypes and also between bortezomib pre-treated and non-treated xenografts (p < 0.05 was considered statistically significant).

### Distribution of doxorubicin in tumours

The distribution of doxorubicin was studied in xenografts measuring 5-8 mm in diameter with and without prior treatment with bortezomib, as described previously [8]. Bortezomib (1 mg/kg) or diluent were administered i.p. 72 h prior to doxorubicin, which was injected i.v. at a dose of 30 mg/kg to facilitate fluorescence detection. Animals were killed 10-15 min post doxorubicin injection and tumours were excised and placed immediately in optimum cutting temperature compound, frozen in liquid nitrogen, and stored at -70 °C prior to sectioning and immunohistochemical staining. Two 10 μm-thick cryostat sections were cut from each tumour (sections ~50 μm apart), mounted on glass slides and air dried.

Doxorubicin fluorescence (which might include a component from fluorescent metabolites) was detected using an Olympus Upright BX50 microscope with a Photometrics Coolsnap HQ2 camera and a 100 W HBO mercury light source equipped with 530-560 nm excitation and 573-647 nm emission wavelength filter sets. Tissue sections were tiled using a motorized stage. Blood vessels in tissue sections were recognized by expression of CD31 on endothelial cells. After imaging for doxorubicin, tissue sections were fixed in acetone, washed in PBS, and blocked with a protein-blocking reagent (ID Labs, Inc., London, ON, Canada). Tissue sections were then stained with a rat anti-CD31 (1/100) antibody for one hour in a humidified chamber, washed in PBS and stained with a Cy3-conjugated goat anti-rat IgG secondary antibody (1/400). CD31-stained sections were re-imaged using the same method used to capture doxorubicin fluorescence.

Composite images were generated by overlaying those for doxorubicin and blood vessels using Media Cybernetics Image Pro Plus Software (Version 6.0). Doxorubicin staining was converted to an 8-bit grey-scale with fluorescence intensities ranging from 1-254, while blood vessels stained with anti-CD31 were represented by an intensity of 255. Regions for data analysis were selected by excluding artefact, fluorescence, and necrosis, and objects <5 μm in diameter were removed. Readings from regions without nuclear staining provided average background fluorescence for each tumour section. The pixel area was 0.4 μm^2^, and the distance to the nearest blood vessel for each pixel within a selected area of interest (AOI) was measured by customized algorithms. Doxorubicin intensity (I) relative to background was averaged over all pixels at a given distance (L) from the nearest blood vessel and plotted as a function of that distance. Doxorubicin distribution in each AOI was determined by calculation of the area under the intensity vs. distance graph and differences between cells lines and treatments were assessed using a *t*-test (p < 0.05 was considered statistically significant).

## Results

### Modification of packing density and drug penetration by EGTA

**Figure 1:**
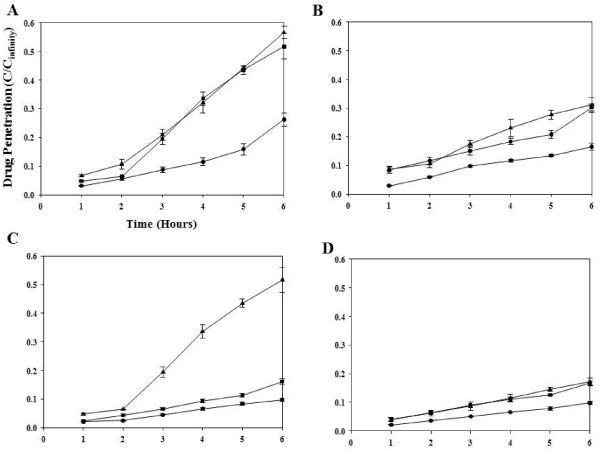
**Effects of EGTA (10 mM) or bortezomib (1uM) pre-treatment on penetration of gemcitabine (panels A and C) and 5-Fluorouracil (panels B and D) through MCC derived from HCT-8Ea (panels A and B) and HCT-8E11 cell lines (panels C and D).** Data are presented as the ratio of radio-labeled drug concentration in compartment 2 to expected drug concentration at equilibrium. Panels shows penetration of gemcitabine or 5-fluorouracil through MCC without pre-treatment (·), and following pre-treatment with EGTA (▲) or bortezomib (■). Data points represent the mean of 3 or more experiments ± SEM.

The calcium chelating agent EGTA was used to disrupt E-cadherin mediated cell-cell adhesion in MCC. To determine the optimal treatment conditions for inducing a significant change in packing density with little or no toxicity, MCC derived from HCT-8Ea and E11 cell lines were pre-treated with 5, 10, or 50 mM of EGTA for 30, 60, or 90 min. As determined by clonogenic assays, exposure of MCC to 10 mM EGTA for 90 min caused no toxicity to either cell line; at this dose mean packing density (+/-SD) was reduced from 50 ± 7.3% to 40 ± 1.6% and from 40 ± 6.8% to 31 ± 4.5%, in MCC derived from HCT-8Ea and HCT-8E11 cells respectively (p = 2.2 × 10^-9^ and p = 8.1 × 10^-6^ for HCT-8Ea and HCT-8E11 cells respectively). The changes in packing density were uniform throughout the MCC and not limited to the MCC surface (p = 0.78 and p = 0.82 for HCT-8Ea and HCT-8E11 cells respectively). The penetration of all anticancer agents was greater in EGTA-modified than in control MCC. EGTA pre-treatment improved penetration of 5-fluorouracil and gemcitabine (measured at 6 h) by ~3.3-4.4 fold in MCC derived from HCT-8Ea cells and by ~1.5-1.9 fold in MCC derived from HCT-8E11 cells (Figure 1). EGTA also led to ~2-fold increase in penetration of doxorubicin (p = 0.02 for HCT-8E11, p = 0.002 for HCT-8Ea; data not shown).

### Bortezomib treatment, packing density and cytotoxicity

We did not observe significant alterations in cellular packing density in MCC derived from HCT-8 colon carcinoma cell lines using sub-cytotoxic doses of bortezomib, but doses that induced less than 50% cell kill decreased cellular packing density (Table 1). Furthermore, assessment of packing density by partitioning of the DAPI-stained MCC images into three equal horizontal image sections showed the changes in packing density to be uniform throughout the MCC (p = 0.86 and p = 0.73 for HCT-8Ea and HCT-8E11 cells respectively). Cells treated in monolayer for 24 h were more sensitive to bortezomib-induced cytotoxicity than those treated in MCC: a one-log cell kill was achieved in monolayer with 250nM and 500nM for HCT-8Ea and HCT-8E11 cells respectively, while similar cytotoxicity in MCC required bortezomib doses greater than 2.5 μM.

**Table 1:** Change in packing density of MCC (mean +/– standard deviation) at 24 h after exposure to varying concentrations of bortezomib

Bortezomib concentration (μM)	MCC Packing Density (% nuclear area)	
	HCT-8Ea	HCT-8E11
0	53.2 ± 5.5	50.8 ± 3.8
0.25	50.9 ± 6.2	48.6 ± 3.2
0.50	50.2 ± 5.6	46.6 ± 4.1
1.0	44.6 ± 5.7*	42.5 ± 3.8*
2.5	45.9 ± 5.6*	MCC disaggregated

MCC derived from both cell lines disaggregate when treated with 5 μM of bortezomib. Packing density changes were shown to be significant after pre-treatment with 1 μM of bortezomib (p = 1.3 × 10^-7^ and p = 1.8 × 10^-6^ for HCT-8Ea and HCT-8E11 derived MCC respectively as assessed using a *t*-test).

* p≤0.05 for comparison with control conditions.

### Bortezomib and penetration of other anti-cancer drugs

For studies of drug penetration, MCC were exposed for 24 h to 1 μM bortezomib or its diluent; this dose induced modest cell kill and led to the greatest change in packing density. Bortezomib pre-treatment improved penetration of 5-fluorouracil by ~1.7-fold and gemcitabine by ~3-fold in MCC derived from HCT-8Ea cells and increased penetration of both drugs by ~2-fold in MCC derived from HCT-8E11 cells at 6 h (Figure 1). Penetration of doxorubicin was also increased by about 1.8-fold at 6 h (p = 0.02 for HCT-8Ea, p = 0.005 for HCT-8E11, data not shown).

### Influence of bortezomib on sensitivity to other anticancer drugs

**Figure 2:**
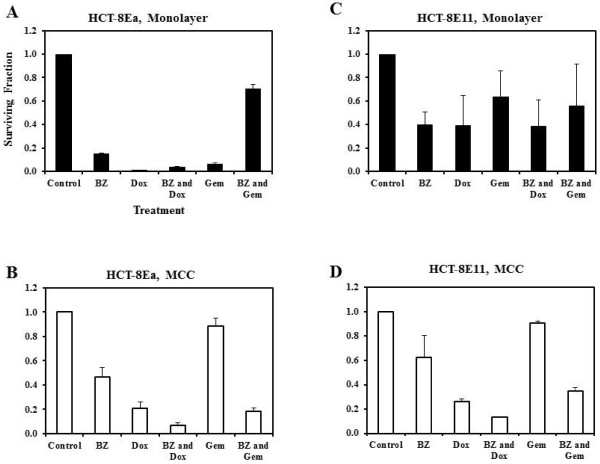
**Influence of bortezomib pre-treatment on sensitivity to doxorubicin or gemcitabine in monolayers (panels A and C) or MCC (panels B and D) derived from HCT-8Ea (panels A and B) or HCT-8E11cells (panels C and D).** Monolayers and MCC were pre-treated with 250 nm or 1 μM bortezomib (BZ) or diluents for 24 h respectively, followed by treatment with doxorubicin (Dox, 10 μM in monolayer or 100 μM in MCC) or gemcitabine (Gem, 20 μM in monolayer or 125 μM in MCC) for 24 h. Data represent the ratio of the number of colonies measured in a given treatment condition to colonies in the untreated condition (mean values from 3 experiments). Bortezomib pre-treatment decreased the cytotoxicity of doxorubicin and gemcitabine for HCT-8Ea cells in monolayer (p = 0.012 and p = 0.001 respectively) but had no significant affect on cytotoxicity of either drug for HCT-8E11 cells (Figure 2, panels **A** and **C**).

Monolayer cultures and MCC were treated with either 10 μM of doxorubicin or 20 μM of gemcitabine for 24 h, with or without a 24-h pre-treatment with bortezomib (250nM in monolayer and 1 μM in MCC). Bortezomib pre-treatment decreased the cytotoxicity of doxorubicin and gemcitabine for HCT-8Ea cells in monolayer (p = 0.012 and p = 0.001 respectively) but had no significant affect on cytotoxicity of either drug for HCT-8E11 cells (Figure 2, panels A and C). ANOVA conducted for monolayer and MCC derived from HCT-8 Ea and E11 cell lines show significant influence on cytotoxicity for bortezomib pre-treatment followed by doxorubicin or gemcitabine treatment (p = 0.018 and p = 0.034 respectively). Post-hoc analysis using the Newman-Keuls Multiple Comparison Test showed significant differences between monolayer and MCC cultures with respect to pre-treatment with bortezomib; bortezomib pre-treatment was shown to enhance doxorubicin and gemcitabine cytotoxicity significantly in MCC and not in monolayer cultures.

### Packing density and interstitial fluid pressure in xenografts

Significant differences in cellular packing density were observed between HCT-8Ea and RA xenografts (68 ± 2.4 vs. 53 ± 4.0, p = 0.003), and between HCT-8E11 and 1R1 xenografts (66 ± 1.9 vs. 59 ± 4.2, p = 0.01). Pre-treatment with bortezomib did not have a significant effect on cellular packing density in xenografts (Table 2). Mean interstitial fluid pressure was higher in xenografts grown from the epithelioid cell lines (9.5 ± 1.4 mmHg in HCT-8Ea vs. 6.7 ± 1.9 mmHg in Ra, p = 0.0005; 10.0 ± 3.5 mmHg in HCT-8 E11 vs. 5.0 ± 1.1 mmHg in 1R1, p = 0.00001). A significant decrease in IFP was observed in HCT-8E11 xenografts at 72 h after treatment with 1.0 mg/kg of bortezomib (p = 0.0006), with a non-significant trend to reduce IFP in HCT-8Ea xenografts (p = 0.053) (Table 2). Microvascular density was shown to be greater in the HCT-8Ra than HCT-8 Ea xenografts (p < 0.0001); in contrast, MVD was greater in the HCT-8E11 derived xenografts than the HCT-81R1 tumours (p < 0.0001; Table 3). A non significant trend towards reducing MVD was shown in HCT-8 E11 and HCT-8Ea xenografts treated with bortezomib (p = 0.1 and p = 0.6 respectively). Bortezomib pre-treatment did not appear to change blood vessel morphology as the gross morphology of tumor xenografts derived from HCT-8Ea remained short and thin post treatment while HCT-8E11 vessels maintained their long and thin morphology.

**Table 2:** Packing density (expressed as the percentage of total area occupied by cell nuclei) and interstital fluid pressure (IFP) in tumour xenografts and the effect of treatment with bortezomib

Bortezomib Treatment Time and Dose	HCT-8Ea xenografts		HCT-8E11 xenografts	
	Packing Density	IFP	Packing Density	IFP
No Treatment	68.3 ± 2.4	9.5 ± 1.4	66.2 ± 1.9	10.0 ± 3.5
24 h after 0.5 mg/kg	68.8 ± 1.7	9.2 ± 3.7	64.5 ± 4.7	7.1 ± 2.1
24 h after 1.0 mg/kg	68.8 ± 3.4	7.3 ± 1.1	65.3 ± 3.3	7.1 ± 3.2
72 h after 0.5 mg/kg	68.2 ± 2.1	8.8 ± 2.5	66.3 ± 2.8	5.8 ± 2.4
72 h after 1.0 mg/kg	67.8 ± 2.9	6.6 ± 2.3	62.1 ± 4.8	5.1 ± 1.8^*^

Data indicate mean +/- standard deviation for 7-9 animals per treatment group.

* p≤0.05 for comparison with control conditions.

**Table 3:** Mean Doxorubicin distribution in xenografts

Tumour type and treatment	Distance from blood vessel at which fluorescence falls to 50% its original value (L)	Doxorubicin distribution (μm × I)	Microvascular Density
HCT-8Ea	24 ± 5	785 ± 190	3.0 ±0.5
HCT-8Ra	31 ± 6	1476 ± 370^*^	6.0 ± 0.8
HCT-8Ea & Bortezomib pre-treatment	29 ±7	1140 ± 190^*^	2.6 ± 0.3
HCT-8E11	29 ± 8	990 ± 220	4.1 ± 0.4
HCT-81R1	43 ± 11	2097 ± 248^**^	2.8 ± 0.2
HCT-8E11 & Bortezomib pre-treatment	35 ± 9	1170 ± 200^**^	3.8 ± 0.6

Drug distribution is represented by the area under the curve representing doxorubicin intensity vs. distance from the nearest blood vessel up to 100 μm. Data were obtained from 10-12 animals and represent mean ± packing density. The distance from blood vessel at which fluorescence falls to 50% its original value (L), presented as mean ± standard deviation was significantly greater in the HCT-8 Ra and 1R1 xenografts than that observed in HCT-8 Ea and E11 tumours (p = 0.003 for HCT-8Ra and Ea and p = 0.008 for HCT-81R1 and E11 tumour xenografts; paired T-tests). This distance was greater in tumor xenografts pre-treated with bortezomib (p = 0.035 and p = 0.048 in HCT-8Ea and HCT-8E11 tumour xenografts pre-treated with bortezomib) than in control.

* p≤0.05 for comparison with HCT-8Ea tumor xenografts.

** p≤0.05 for comparison with HCT-8E11 tumor xenografts.

### Effects of bortezomib on distribution of doxorubicin in xenografts

**Figure 3:**
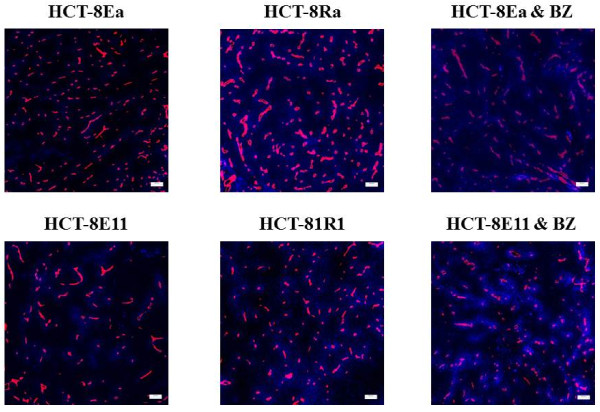
Composite colour images of doxorubicin fluorescence (pseudo-coloured blue) relative to blood vessels (pseudo-coloured red) in HCT-8 Ea and Ra, HCT-8E11 and 1R1 tumour xenografts pre-treated with saline, and for HCT-8Ea and E11 xenografts pre-treated with 1 mg/kg bortezomib (BZ) 72 h prior to doxorubicin administration. Bar, 100 μm.

**Figure 4:**
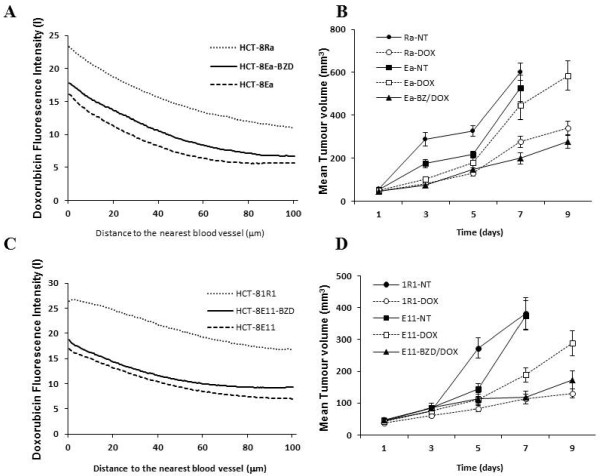
**Intensity of doxorubicin fluorescence (I) plotted against distance from the nearest blood vessel (μm) is shown for HCT-8Ea and Ra (A) and HCT-8E11/1R1 (C) xenografts. Mean values are shown for ~160 areas of interest from 20 tumours in 10 mice.** Upper curves represent drug penetration through HCT-8R tumors, middle curves represent pre-treatment with bortezomib, while lower curves represent controls. Growth curves for HCT-8Ea/Ra (**B**) and HCT-8E11/1R1 (**D**) xenografts following single treatments with doxorubicin (8 mg/kg) with or without prior bortezomib (1 mg/kg). Greater growth delay was observed for the loosely packed HCT-81R1 tumour xenografts than their tightly packed HCT-8E11 counterparts (p = 0.0012) with similar results for HCT-8Ra and HCT-8Ea derived xenografts (p = 0.004). Bortezomib pre-treatment did not significantly enhance doxorubicin treatment efficacy in tumour xenografts derived from HCT-8Ea and E11 cell lines (p = 0.45 and p = 0.09 respectively). Data represent means and SEM for 10-12 mice.

Composite colour images of doxorubicin relative to blood vessels in xenografts are shown in Figure 3. As reported previously [8], doxorubicin fluorescence decreased with increasing distance from blood vessels (Figures 4 A and 4 C). The overall drug availability in areas of interest was measured by the area under the fluorescence intensity/distance curve up to 100 μm (Table 3). The distance at which background-subtracted doxorubicin fluorescent intensity decreased to half its original value (L) was greater in HCT-8 Ra and 1R1 xenografts than that in HCT-8Ea and E11 tumours. Doxorubicin distribution in the xenografts derived from the loosely-packed HCT-8R sub-lines was approximately 2-fold greater than that observed in the corresponding epithelioid HCT-8E sub-lines. Pre-treatment with bortezomib enhanced doxorubicin distribution in HCT-8Ea (n = 9, p = 0.024) and HCT-8E11 (n = 12, p = 0.054) xenografts (Figures 3,4 A and 4 C, Table 3).

### Effect of drugs on tumour growth

No significant differences in tumour growth were observed between xenografts derived from epithelioid and round cell variants without treatment. All sub-lines were rather resistant to maximal tolerated doses of doxorubicin; however, greater delay in growth was observed for the loosely packed HCT-81R1 tumour xenografts than their tightly packed HCT-8E11 counterparts (p < 0.001). Similar patterns of tumour growth delay were observed between HCT-8Ra and HCT-8Ea derived xenografts (p = 0.004) (Figures 4 B & D). Neither bortezomib nor doxorubicin treatment alone influenced growth of HCT-8Ea and HCT-8E11 xenografts (based on data after 11 days). A trend towards reduced tumour growth rates was observed in animals receiving bortezomib 72 h prior to doxorubicin compared to those treated with doxorubicin alone. Doxorubicin treatment (alone or following bortezomib) was associated with 10-15% weight loss.

## Discussion and conclusions

The present study shows that pre-treatment with the proteasome inhibitor bortezomib can decrease packing density in MCC, a tissue culture model for solid tumours, and improve the penetration of other drugs through them. Furthermore, through quantification of the distribution of doxorubicin in tumour xenografts, we show better drug distribution and cytotoxicity in tumours derived from loosely packed as compared to tightly packed sub-lines of HCT-8 human colon carcinoma. Bortezomib was also able to modify the distribution of doxorubicin in the tightly packed HCT-8 xenografts, probably by reducing IFP.

Interactions between tumour cells and components of the ECM can protect solid tumours from toxic stimuli [30-33]. Agents that modify or disrupt cell-cell or cell-matrix adhesion have been used to overcome cell adhesion-mediated drug resistance (CAM-DR) in multi-cellular spheroids and xenografts. Hyaluronidase decreased resistance of multi-cellular spheroids to several anticancer drugs including paclitaxel, doxorubicin, and vinblastine [15,34], while antibodies against E-cadherin or β_1_-integrin were shown to increase drug sensitivity in spheroids or solid tumours [16,31,35]. Our previous studies have shown greater drug penetration and efficacy in MCC derived from colon carcinoma cell lines with a defect in alpha-E-catenin and lack of adherens junctions [3]. Our analyses of doxorubicin distribution and growth delay of tumour xenografts also show significant differences in drug distrubition in epithelioid and round cell pairs of cell lines, where the penetration of chemotherapeutic agents was greater through MCC derived from loosely packed than the tightly packed sub-lines. The AUC for doxorubicin distribution in the loosely packed HCT-81R1 and Ra xenografts was approximately 2-fold greater than that observed in the tightly-packed HCT-8Ea and HCT-8E11 tumours. Differences in tumour drug distribution as a function of packing density have also been reported by Au et al. (Kuh et al., 1999; Zheng et al., 2001): high cell density was shown to reduce the penetration of doxorubicin and paclitaxel in PC3 and human pharynx FaDu tumours in histoculture and *in vivo*.

To assess the ability of anti-adhesive agents to modify drug penetration, we conducted proof of principle experiments in MCC, using the calcium chelating agent EGTA, which disrupts E-cadherin mediated cell adhesion. Pre-treatment with non-toxic doses of EGTA significantly decreased cellular packing density, and led to improvement in drug penetration in MCC derived from colon carcinoma cell lines.

Bortezomib has been reported to alter the adherence of multiple myeloma cells to ECM proteins and bone marrow stromal cells [36], and to disrupt cell adhesion in spheroids derived from an ovarian cancer cell line [24]. Bortezomib was also shown to reduce cell adhesion in squamous cell cancer by down-regulation of the desmosomal cadherin Dsg-2 [37]. These studies provided the rationale for evaluation of bortezomib as a modifier of cell-cell adhesion and cellular packing density in solid tumours.

In our studies, reduction of cellular packing density in MCC might be due either to cell killing by bortezomib or to reduced cell adhesion (or both); enhanced drug penetration is most likely related to reduced packing density although we cannot exclude some effect due to loss of cells from the surface of the MCC after drug treatment. Interactions between tumour cells and components of the extracellular matrix (ECM) have been shown to protect solid tumours from a number of apoptotic stimuli, and agents that modify or disrupt cell-cell or cell-matrix adhesion have been used successfully to overcome cell adhesion-mediated drug resistance (CAM-DR) in multicellular spheroids and xenografts established from a variety of tumours. Previous studies by Au and colleagues (Kuh et al., 1999; Zheng et al., 2001) have shown increased penetration of paclitaxel and doxorubicin following a low-dose pre-treatment with these drugs, and they attributed this to induction of apoptosis and the subsequent increase in interstitial space. Several studies have reported enhanced sensitivity to chemotherapy after bortezomib treatment, although underlying mechanisms have not been elucidated [17-19,22,38,39]. The anticancer drugs chosen in our study ranged from ineffective (5-fluorouracil), minimally cytotoxic (gemcitabine), to moderately cytotoxic (doxorubicin) for HCT-8 cells in culture. Bortezomib pre-treatment either reduced or did not change the cytotoxicity of these drugs in monolayer cultures, while it enhanced cytotoxicity in MCC; this result suggests strongly that the effects of bortezomib to influence sensitivity to other drugs is dependent on cell contact.

Drugs are transported from the circulatory system into the interstitial space both by diffusion and by convection. Elevated interstitial fluid pressure has been shown to limit the efficacy of anticancer drugs by reducing trans-capillary transport and tissue penetration by convection [40-42]. Agents that reduce tumour IFP have been shown to improve drug distribution and efficacy in pre-clinical and clinical settings [43-45]. Agents that reduce IFP have been shown to enhance the transcapillary transport of low molecular weight tracers in experimental rat colon cancer models and NSCLC xenografts and increase the penetration of monoclonal antibodies in various tumour xenografts [44,46]. We observed a correlation between cellular packing density and IFP in xenografts derived from HCT-8 sub-lines. Previous studies have reported that high cell density around blood vessels can lead to elevated IFP. It has also been shown that a reduction in tumour cell density following treatment with paclitaxel (due to the induction of apoptosis) can decrease IFP. We observed no significant reduction in cellular packing density in colon carcinoma xenografts but there was a reduction in IFP at 72 h after bortezomib administration, and this was associated with improvement in doxorubicin penetration. The reduction in IFP after bortezomib treatment cannot be attributed to a reduction in MVD, as bortezomib did not demonstrate a significant anti-angiogenic activity in HCT-8 derived tumor xenografts. Furthermore, tumor xenografts derived from the HCT-8Ea cell line showed lower MVD than the HCT-8Ra tumor xenografts, yet exhibited significantly greater IFP. The xenografts that were evaluated are quite resistant to anticancer drugs. Our data show greater doxorubicin cytotoxicity in the loosely packed HCT-8Ra and HCT-81R1 than the tightly packed HCT8-Ea and HCT8-E11 xenografts. These data are similar to our observations of doxorubicin cytotoxicity and drug penetration using the MCC model and provide further evidence for the role of tumour physiology and drug penetration in drug resistance. In addition, our results further support the use of the MCC model in assessing the role of tumour physiology and architecture in chemotherapeutic resistance. Our findings suggest a trend towards greater growth suppression in tumours treated with bortezomib prior to doxorubicin treatment than those treated with doxorubicin alone, but future studies will benefit from using other drugs to which HCT-8 tumours show greater sensitivity or other tumours that are more sensitive to doxorubicin, in order to assess bortezomib’s potential as a chemo-sensitizer.

In summary, we have provided further evidence for the role of tumour micro-environment in contributing to impaired drug distribution and cytotoxicity in solid tumours. In addition, our studies show that bortezomib can modify the microenvironment and enhance drug penetration in xenografts; its potential to enhance the effects of other anticancer drugs for treatment of solid tumours merits further investigation.

## Competing interests

The authors declare that they have no competing interests.

## Author’s contributions

RG participated in designing, planning and carrying out *in vitro* and *in vivo* drug distribution studies, imaging, analysis and growth delay studies as well as drafting and revising the manuscript. IFT conceived the concepts underlying the study, designed the experiments, revised the manuscript and provided funding, grant support and oversight. Both authors read and approved the final manuscript.

## Pre-publication history

The pre-publication history for this paper can be accessed here:

http://www.biomedcentral.com/1471-2407/12/214/prepub
